# Development of Fearfulness in Birds: Genetic Factors Modulate Non-Genetic Maternal Influences

**DOI:** 10.1371/journal.pone.0014604

**Published:** 2011-01-27

**Authors:** Cécilia Houdelier, Sophie Lumineau, Aline Bertin, Floriane Guibert, Emmanuel De Margerie, Matthieu Augery, Marie-Annick Richard-Yris

**Affiliations:** 1 UMR-CNRS 6552 Ethologie animale et humaine, Université Rennes 1, Rennes, France; 2 UMR 85- Physiologie de la reproduction et des comportements - INRA-CNRS-Université de Tours-Haras Nationaux, Nouzilly, France; Pennsylvania State University, United States of America

## Abstract

The development of fearfulness and the capacity of animals to cope with stressful events are particularly sensitive to early experience with mothers in a wide range of species. However, intrinsic characteristics of young animals can modulate maternal influence. This study evaluated the effect of intrinsic fearfulness on non-genetic maternal influence. Quail chicks, divergently selected for either higher (LTI) or lower fearfulness (STI) and from a control line (C), were cross-fostered by LTI or STI mothers. Behavioural tests estimated the chicks' emotional profiles after separation from the mother. Whatever their genotype, the fearfulness of chicks adopted by LTI mothers was higher than that of chicks adopted by STI mothers. However, genetic background affected the strength of maternal effects: the least emotional chicks (STI) were the least affected by early experience with mothers. We demonstrated that young animal's intrinsic fearfulness affects strongly their sensitivity to non-genetic maternal influences. A young animal's behavioural characteristics play a fundamental role in its own behavioural development processes.

## Introduction

Many animal species present interindividual behavioural and/or physiological differences that are consistent over age and context, and have been labelled temperament, coping strategies, styles, syndromes or personality traits [1]–[3]. Fearfulness (or emotional reactivity) is defined as the propensity to be frightened more or less easily [4]. This is one of the traits of temperament that implies the predisposition of an individual to respond similarly to a variety of potentially alarming challenges [2]. This trait is a major characteristic of animals including humans as it determines the ability of individuals to cope with stressful events throughout their lives.

Recent investigations have contributed to the growing appreciation of non-genetic maternal influences on offspring's phenotypic outcomes, and particularly their emotional outcomes. Mothers transfer a variety of non-genetic factors to their offspring; these include neurobiological traits (DNA methylation patterns, chromatin marking systems, hormones) [5], [6], behavioural characteristics (emotive and social traits, sexual and maternal behaviour, endogenous rhythms) [7]–[11], and a range of sensory stimulations necessary for normal development. Understanding how these developmental resources contribute to the emergence, maintenance, or modification of phenotypic traits has received increasing attention, particularly in neuroendocrinology and genetic research [12], [13].

However, far less is known about the sensitivity of young organisms to maternal influence. Yet, non-genetic maternal influence can vary in relation to their offspring's gender. Thus, adoptive mice mothers affect the development of fearfulness in their male, but not in their female, offspring [14]. Maternal effects on the development of mammals' and birds' social or sexual preferences appear stronger in male than in female offspring [8], [9]. Anisman *et al.* [15] reported a difference in sensitivity of young mice to maternal effects in relation to genetic origin: pups from the higher emotional reactivity line were influenced more by maternal care than were those from the lesser reactive line. Thus, genetic factors of young can influence dam-pup interactive styles and can affect their future responses to subsequent stressors.

Here, we investigated how genetic factors modulate mother effects on the behavioural development of young quail. Birds are interesting models for investigating behavioural maternal transmission mechanisms as chicks develop physiologically independently of their mothers, and contrary to mammals, maternal chemical compounds cannot be transmitted to young via milk. Japanese quail (*Coturnix coturnix japonica*) is a precocial species and maternal care lasts only 11 days [16]. An original procedure of maternal behaviour induction developed specifically for gallinaceous species [17] facilitates experimental control of the influence of genetic and non-genetic factors. Finally, the emotional traits of Japanese quail are determined partly by genetic factors [18], as well as by early maternal influence [19], [20].

In this study, we used two genetic lines of quail selected on their duration of tonic immobility (TI) [18]. Tonic immobility is an involuntary, reflexive response to fear-inducing stimuli, present in invertebrate and vertebrate species [21]. This behavioural response has been used to a great extent as an index of fearfulness in poultry [22]–[24]. Quail from the LTI (Long Tonic Immobility) line were selected for a long TI duration whereas birds from the STI (Short Tonic Immobility) line were selected for a short TI duration [18]. This selection program modified general underlying fearfulness rather than exerted specific effects on TI [25]. LTI line quail took longer to emerge into a novel environment, expressed there more freezing behaviour and less explorations [25], [26]. Their latencies to approach novel food were longer and they were more disturbed by a sudden introduction of a frightening stimulus into their home-cage [27] or in the presence of humans [26]. LTI quail are considered to have high levels of fearfulness and STI quail to have low levels of fearfulness.

So, in this study, we hypothesised that intrinsic fearfulness of LTI and STI chicks would affect non-genetic maternal effects by modulating mothers' impacts on the behavioural development of their offspring.

## Materials and Methods

### Ethics Statement

All the animal care and research involved was approved by the departmental direction of veterinary services (Ille et Vilaine, Permit number 005283) in accordance with the European Communities Council Directive of 24 November 1986 (86/609/EEC).

### Animals

Mills & Faure [18] described in detail the selection procedures used to develop LTI and STI lines. Briefly, birds of two commercial strains where reciprocally crossed so as to constitute a common base line population for the two selected lines. TI was estimated when chicks were 9 to 10 days old. TI duration was defined as the time when an unrestrained chick remained immobile after 10 s of manual restraint. The maximum number of induction (NI) allowed to induce TI was limited to five, and TI was limited to 300 s. When TI could not be induced after five attempts the bird was deemed to be unsusceptible and given score of NI  = 5 and TI = 0 s. When a bird failed to right itself after 300 s, it was given scores of NI between 1 and 5, and TI = 300 s [22].

Adult females and chicks used in this study belonged respectively to the 36^th^ and 37^th^ generations of these genetic lines. A control line was also used, characterised by an intermediate TI level. All birds came from the INRA UEPEAT experimental unit (1295), Nouzilly, France.

### Cross-fostering design and housing

For each experiment, maternal behaviour was induced in 22 STI and 21 LTI adult female quail via an original procedure facilitating rapid emergence of maternal care [17], [26]. Chicks arrived in the laboratory a few hours after they had hatched and were wing banded. At the beginning of a dark phase, three chicks were placed underneath each female in a nest box and then each box was shut up for the night. Tactile and auditory stimulations emitted by chicks induce a rapid emergence of parental responses in females that express the full repertoire of maternal behaviour the following morning [17]. Maternal behaviour includes warming (the female erects her feathers and crouches over her chicks to keep them warm), maternal calls (cooing, a “hoarse peep” and a food call) and brood defence. As LTI females require longer than STI females to develop maternal care during the first day of mothering [17], chicks from a commercial line were used for induction. Females were observed for 5 hours and only those who expressed the full maternal behavioural repertoire were retained (in similar proportions for the two lines) and the commercial chicks were then replaced by three experimental chicks coming from the same genetic line. So, brooded groups were constituted of one female with 3 adopted chicks.

Three successive experiments, involving the same maternal females, were conducted at 5-week intervals under similar conditions of temperature and light/dark cycles: (1) 45 STI chicks were raised by STI mothers (SS chicks) and 32 STI chicks by LTI mothers (SL chicks); (2) 64 control chicks were raised by STI females (CS chicks) and 57 by LTI mothers (CL chicks); (3) 54 LTI chicks were raised by STI females (LS chicks) and 55 by LTI mothers (LL chicks). Previous reports showed that maternal experience had no significant effect on maternal care in domestic hens [28]. Brooding lasts 11 days [16]; mothers were then removed, and young (remained in their sibling group) were tested when they were between 11 and 21 days old.

Most test groups included three young. However, due to mortality, some test groups were composed of two chicks. However, proportions of 2-chick groups and 3-chick groups did not differ between sets for the same session (χ^2^ = 0.49 (LTI chicks); χ^2^ = 2.71 (C chicks); χ^2^ = 0.001(STI chicks), d*f*  = 1, p≥0.10 for the three sessions) or between the three sessions (χ^2^ = 0.33, d*f*  =  2, p>0.80).

As in quail morphological sexual dimorphism appears only around 3 weeks old, both male and female chicks were tested in this experiment. However, sex ratios were not different, either for a session (χ^2^ = 0.044 (LTI chicks); χ^2^ = 0.57 (C chicks); χ^2^ = 0.42(STI chicks), d*f*  =  1, p>0.30 for the three sessions): numbers of males and females were similar (mean sex ratio (N males/N females)  =  1.02±0.08), or between the three sessions (χ^2^ = 0.97, d*f*  = 2, p>0.50).

Brooded groups and young groups (after separation from mother) were housed in the same room, in wire-mesh cages (51×40×35 cm) with opaque lateral walls (preventing visual contacts between brooded groups). Each cage contained a drinker, a feeder and a nest box. Water and food were available ad libitum. A 10∶14 hr light:dark cycle and an ambient temperature of 20±1°C were maintained.

### Procedure and tests

Ethological tests, used for poultry, presenting different potentially fearful situations were used to assess the fearfulness of chicks [29]. Indeed, fearfulness is a complex trait and a combination of behavioural tests mainly aiming to induce a state of fear is usually needed to assess the susceptibility of individuals. Chicks were tested after separation from their mothers to avoid disrupting maternal behaviour. The same person performed all the tests and always wore the same clothes.

#### i) Reactivity to humans

*1. Human-observer test*: the experimenter passed (walked slowly) in front of each home cage (approximately 40 cm from the cage door) at 6-min intervals during two 96-min periods, one in the morning and one in the afternoon (a total of 32 scans per cage). Every 6 min, the experimenter recorded instantaneously the number of birds expressing behaviours known to reflect fear that we subsequently called fear behaviours: active fear behaviours include withdrawal (quail move away from the experimenter), and/or violent attempts to escape (quail run about in the cage and jump violently); passive fear behaviours corresponded to behavioural inhibition (low postures corresponding to observations when animals are lying down or crouching, expressed in relation to all observation's postures) [4], [30]. The experimenter also recorded comfort activities (exploration, feeding, preening and resting) that reflect a low level of fear in birds. Other behaviours that do not reflect a particular high or low level of fear were also noted (observation's postures, walk). Quail were tested when they were 19 days old.

*2. Hand-on-home-cage-door-test:* The procedure of this test was similar to that described for the previous test, but each time the experimenter passed in front of a cage, he placed one hand on the door for 1s and recorded the bird's immediate reaction: active and passive fear behaviours, comfort activities and other observations postures or walk. Quail were tested when they were 21 days old.

In these two procedures, birds were tested in their group and in their home-cage. Opaque lateral walls of the cages prevented visual contacts between groups and thereby, any possible effect of a group on the reactions of the neighbouring groups. A particular testing order was used to prevent the birds from seeing the experimenter just before their test.

#### (ii) Non-specific fearfulness

These tests were carried out in environments that were novel for the chicks. Chicks were caught before each test and carried gently in a wooden box (10×10×10 cm) to the test room. Although these tests involved some contact with the experimenter, cross-test correlations were consistent with other within and between test correlations reported for domestic hens [24], Japanese quail [31] and mammals [29], clearly supporting the notion that these tests revealed general, non-specific fearfulness rather than only stimulus-specific responses.

*1. Tonic Immobility test*. This protocol was similar to that previously described in the 2^nd^ paragraph of this part. TI induction numbers and TI duration were evaluated in 12-day old birds. TI duration is positively correlated to an animal's fear level [32].

*2. Emergence test*. This test followed a protocol similar to that described by Jones *et al.* [25]. Quail were placed in an opaque wooden box. This box was placed at the entrance of a larger well-lighted experimental box (43×40×48 cm) equipped with an observation window. The transport box was kept closed for 1 min and then left opened for 3 min. The experimenter noted latency of emergence from the wooden box into the experimental box. This parameter is a good estimate of fearfulness: fearful animals take longer to emerge [31], [33]. When a quail had not emerged, a maximum score of 180 s was recorded. When a quail emerged from the box, the experimenter noted its comfort activities and active and passive fear behaviours. Young were tested when they were 14–15 days old.

*3. Open-field test*. Quail were placed individually in the middle of a wire-netting cylinder (120 cm diameter ×62 cm height) on a linoleum floor, for 5 min. Hidden behind a black curtain with an observation window, the experimenter recorded latency of first step, comfort activities and active and passive fear behaviours. Subjects were 16–17 days old.

### Statistical analyses

Data for birds from a same mothering session were compared statistically. For tests performed on chicks' brood (hand-on-home-cage-door test and human-observer test), frequencies of each behavioural item were weighted by the number of chicks presented (frequency per individual) and used to compare data within a given session (N chicks groups: 21 CL vs 22 CS, 20 LL vs 19 LS, 11 SL vs 16 SS). Synchronisation of siblings' responses to human perturbation was evaluated for hand-on-home-cage-door-test. For that, we compared the frequency of observations (or scans) when at least two of the three chicks performed the same behaviour (active fear behaviour, passive fear behaviour or comfort behaviour) and also the frequency of observations when all chicks were close together (in the same half of home cage). Only data from cages containing three chicks were analysed (N chicks groups: 15 CL vs 20 CS, 15 LL vs 16 LS, 9 SL vs 13 SS). Data from individual tests were used to analyse maternal effect: latencies of behaviour, tonic immobility scores, total occurrence (active fear behaviour, comfort activities) and relative frequencies (passive fear behaviour) (N chicks: 57 CL vs 64 CS, 55 LL vs 54 LS, 45 SL vs 32 SS).

Kolmogorov–Smirnov tests were used to determine whether data sets were normally distributed. As data were not all normally distributed, Mann–Whitney U-tests with Bonferroni corrections for multiple comparisons were used. Chi-square tests compared numbers of significant statistical differences observed among lines and mother's types. Frequencies of observations with behavioural synchronisation and with close position were transformed by arc sin square roots and analysed with a two-ways ANOVA and subsequent post-hoc Bonferroni tests. Throughout the text, corrected p-values are reported. Data are presented as means±SEM. All analyses were performed using “Statview 4.5” statistic software (SAS Institute Inc., Cary, USA).

## Results

### Reactivity to humans

The human-observer tests and hand-on-home-cage-door tests revealed that control chicks reared by LTI females (CL) expressed more fear behaviours (both active and passive) in the presence of humans than did chicks reared by STI quail (CS) (Table 1). CL chicks expressed comfort behaviours less frequently than did CS chicks (Table 1).

**Table 1:** Behaviours of control chicks during reactivity-to-humans tests.

				*Mann-Whitney U-test*	
		CL	CS	U value	p
Human-observer test	Active fear Behav	**19.3±3.1**	**1.8±0.8**	**27.5**	**<0.0001**
	Passive fear Behav	**30.9±2.9**	**2.6±0.7**	**1**	**<0.0001**
	Comfort Activities	**10.3±2.1**	**23.3±3.3**	**102**	**0.0017**
Hand-one-home-cage-door test	Active Fear Behav	**34.1±3.9**	**10.9±1.5**	**42.5**	**<0.0001**
	Passive fear Behav	**50.8±3.5**	**4.1±0.6**	**0**	**<0.0001**
	Comfort Activities	**4.9±1.1**	**16.4±2.2**	**65**	**<0.0001**

Frequencies of behaviours (% mean ± SEM per individual), reflecting a high level (active and passive fear behaviours) or a low level (comfort activities) of fear, emitted by birds during the human-observer test and the hand-on-home-cage-door test. Data were weighted by the total number of behaviours emitted during tests.

CL: control young reared by LTI female; CS: control young reared by STI quail.

LTI young reared by LTI females (LL) expressed more fear responses than did LTI chicks reared by STI quail (LS) in front of the experimenter (except for passive fear responses in the hand-on-home-cage-door tests where only a tendency could be evidenced) (Table 2). LL young tended to express less comfort behaviour than did LS chicks (Table 2).

**Table 2:** Behaviours of LTI chicks during reactivity-to-humans tests.

				*Mann-Whitney U-test*	
		LL	LS	U value	p
Human-observer test	Active fear Behav	**18.7±3.1**	**7.2±1.7**	**81**	**0.0023**
	Passive fear Behav	**24.0±2.9**	**9.6±1.6**	**62**	**0.0003**
	Comfort Activities	*20.3±2.9*	*30.5±4.3*	*128.5*	*0.088*
Hand-one-home-cage-door test	Active Fear Behav	**46.2±4.6**	**29.0±4.1**	**101**	**0.013**
	Passive fear Behav	*17.8±3.7*	*9.2±2.1*	*124.5*	*0.069*
	Comfort Activities	*15.3±3.1*	*23.2±4.0*	*128.5*	*0.088*

Frequencies of behaviours (% mean ± SEM per individual), reflecting a high level (active and passive fear behaviours) or a low level (comfort activities) of fear, emitted by birds during the human-observer test and the hand-on-home-cage-door test. Data were weighted by the total number of behaviours emitted during tests.

LL: LTI young reared by LTI female; LS: LTI young reared by STI quail.

Adoptive mother's line induced fewer differences in STI chicks. Although STI chicks reared by LTI females (SL) showed more passive fear behaviours than did STI chicks reared by STI quail (SS) in the hand-on-home-cage-door test, none of the other parameters differed significantly in relation to maternal care (Table 3).

**Table 3:** Behaviours of STI chicks during reactivity-to-humans tests.

				*Mann-Whitney U-test*	
		SL	SS	U value	p
Human-observer test	Active fear Behav	13.5±3.1	10.1±2.5	82.5	0.78
	Passive fear Behav	8.1±2.6	4.2±1.2	63	0.21
	Comfort Activities	26.4±3.7	25.4±3.7	85.5	0.90
Hand-one-home-cage-door test	Active Fear Behav	36.7±6.2	31.0±3.5	72.5	0.44
	Passive fear Behav	**18.0±3.4**	**9.1±1.6**	**41**	**0.02**
	Comfort Activities	13.3±2.6	12.4±1.7	84.5	0.86

Frequencies of behaviours (% mean ± SEM per individual), reflecting a high level (active and passive fear behaviours) or a low level (comfort activities) of fear, emitted by birds during the human-observer test and the hand-on-home-cage-door test. Data were weighted by the total number of behaviours emitted during tests.

SL: STI chicks reared by LTI females; SS: STI chicks reared by STI quail.

**Figure 1:**
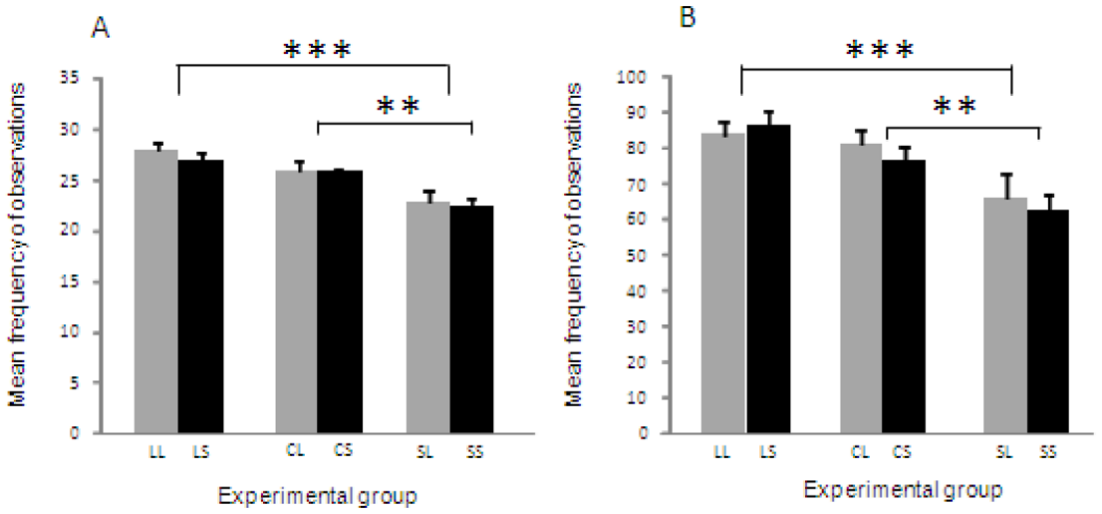
Synchronisation of behaviours (A) and proximity (B) in sibling groups during the hand-on-home-cage-door test. (A) Frequencies of observations (mean ± SEM) when all sibling chicks performed the same behaviour during the test, for each mother group. (B) Frequencies of observations (mean ± SEM) when sibling chicks are close together during the test. LL: LTI chicks reared by LTI females; LS: LTI chicks reared by STI quail. CL: control chicks reared by LTI female; CS: control young reared by STI quail. SL: STI chicks reared by LTI females; SS: STI chicks reared by STI quail. Post-hoc Bonferroni test: ** p<0.01; ***p<0.0001.

During hand-on-home-cage-door tests, the synchronisation of behaviours between sibling chicks appeared to be influenced by their genetic line (two-ways ANOVA, F_2,82_  = 12.424, p<0.0001). However, no effects of mother's line (F_1,82_  = 1.406, p = 0.24) and no interactions between chicks' line and mother's line (F_2,82_ = 0.171, p = 0.84) on this parameter could be evidenced. So, the behavioural responses of S chicks appeared synchronised less often than those of L chicks (post-hoc Bonferroni test, p<0.0001) and of C chicks (post-hoc Bonferroni test, p = 0.0016) (fig.1A). No differences in synchronisation were observed between C and L chicks (post-hoc Bonferroni test, p = 0.0372) (fig.1A). Moreover, the proximity of sibling chicks during the hand-on-home-cage-door test was influenced by the chicks' genetic lines (two-ways ANOVA, F_2,82_ = 7.281, p = 0.0012). However, again, no effects of mothers' line (F_1,82_  = 0.181, p = 0.67) and no interactions between chicks' line and mothers' line (F_2,82_  = 0.272, p = 0.76) on sibling chicks proximity could be evidenced. S chicks appeared less often close together in their cage than did L chicks (post-hoc Bonferroni test, p<0.0001) and C chicks (post-hoc Bonferroni test, p = 0.0004) (fig.1B). No differences in sibling proximity were observed between C and L chicks (post-hoc Bonferroni test, p = 0.20).

### Non-specific fearfulness

**Figure 2:**
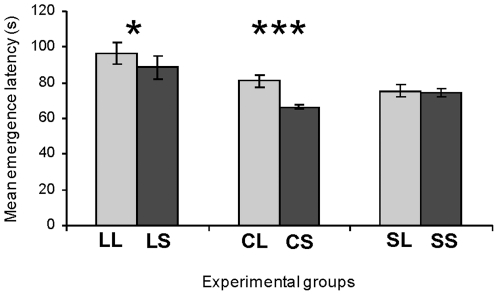
Emergence latency of chicks during the emergence test. Mean ± SEM emergence latencies (s) of chicks reared by LTI and STI mothers. LL: LTI chicks reared by LTI females; LS: LTI chicks reared by STI quail. CL: control chicks reared by LTI female; CS: control young reared by STI quail. SL: STI chicks reared by LTI females; SS: STI chicks reared by STI quail. Mann-Whitney *U-*test: * p<0.05; ***p<0.0001.

**Figure 3:**
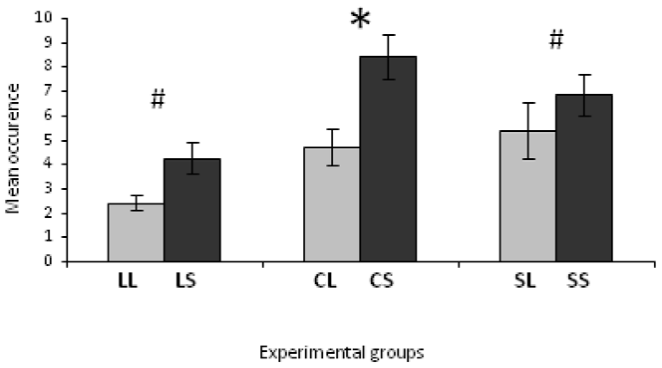
Frequencies of comfort activities of chicks during the open-field test. Mean ± SEM comfort activities, in the open-field test, of chicks reared by LTI and STI mothers. LL: LTI chicks reared by LTI females; LS: LTI chicks reared by STI quail. CL: control chicks reared by LTI female; CS: control young reared by STI quail. SL: STI chicks reared by LTI females; SS: STI chicks reared by STI quail. Mann-Whitney *U-*test: # 0.05<p<0.1; * p<0.05.

Fearfulness of C chicks differed in relation to adoptive mother's line in individual tests. Thus, in the tonic-immobility test, fewer inductions were needed to induce tonic immobility in CL young than in CS chicks (1.4±0.1 vs 1.9±0.1; Mann-Whitney *U*-test, *U* = 1340, p = 0.004), and CL chicks stayed longer in this state than did CS chicks (79.0±6.9 s vs 53.7±5.2 s; Mann-Whitney *U*-test, *U* = 1188, p = 0.001). CL chicks resumed moving later than did CS chicks in a novel environment, in the emergence test (Mann-Whitney *U*-test *U* = 574, p<0.0001) (fig. 2) and also in the open-field test (20.1±3.2 s (CL) vs 6.0±1.5 s (CS); Mann-Whitney *U*-test, *U* = 631, p<0.0001). In these environments, CL chicks showed more passive fear behaviours than CS chicks (open-field test, 3.1±0.9% (CL) vs 0 (CS), Mann-Whitney *U*-test, *U* = 1440, p<0.0001; emergence test, 21.7±3.0(CL) vs 4.0±1.2% (CS), *U*-test, *U* = 796, p<0.0001), but active fear behaviour levels did not differ significantly between CL and CS chicks (open-field test, Mann-Whitney *U*-test, *U* = 1781.5, p = 0.67; emergence test, *U* = 1808, p = 0.92). Finally, CL young expressed comfort behaviour less frequently than did CS chicks in the emergence test (0.68±0.16 (CL) vs 3.39±0.91 (CS); Mann-Whitney *U*-test, *U* = 1303 p = 0.003) as well as in the open-field test (Mann-Whitney *U*-test, *U* = 1072.5, p<0.0001) (fig. 3).

The behaviour of L chicks in individual tests revealed an effect of maternal care, but to a lesser degree than did the behaviour of control young. Neither durations of immobility (Mann-Whitney *U*-test, *U* = 1340, p = 0.23) nor numbers of inductions (Mann-Whitney *U*-test, *U* = 1539.5, p = 0.99) differed significantly between the two sets of chicks in tonic immobility tests. However, LL young started to move later in the open-field test (33.5±5.5 (LL) vs 29.9±6.8 (LS); Mann-Whitney, *U*-test, *U* = 1163, p = 0.035) and came out of their box later in the emergence test than did LS chicks (Mann-Whitney *U*-test, *U* = 1142, 0.037) (fig. 2). In these tests, they also presented more passive fear behaviours than did LS young (open-field test, 7.2±1.8% (LL) vs 2.0±0.6% (LS); Mann-Whitney *U*-test, *U* = 1161, p = 0.011 - emergence test, 23.6±3.3 (LL) vs 9.6±1.6% (LS); Mann-Whitney *U*-test, *U* = 849.5, p = 0.0021), but their active fear behaviours did not differ significantly (open-field test, Mann-Whitney *U*-test, *U* = 1480.5, p = 0.65; emergence test, *U* = 1402, p = 0.42). Finally, LL chicks tended to perform less comfort activities in the open-field than did LS chicks (Mann-Whitney *U*-test, *U* = 1240, p = 0.098) (fig. 3). This tendency was not observed in the emergence test (Mann-Whitney *U*-test, *U* = 1268.5, p = 0.14).

The individual tests revealed no clear influence of adoptive mother's line on S chicks. The two sets of S chicks did not differ significantly in the tonic immobility tests (induction number, Mann-Whitney *U-test*, *U* = 694, p = 0.78; TI duration, *U* = 712.5, p = 0.94). Moreover, latencies to start moving in the open-field and emergence tests did not differ significantly between SL and SS chicks (open-field test, Mann-Whitney *U*-test, *U* = 673, p = 0.61; emergence test, *U* = 685, p = 0.90) (fig. 1). These two sets expressed similar levels of fear behaviours (active and passive) in these environments (open-field test, Mann-Whitney *U*-test, *U* = 705, p = 0.69 (passive fear behaviour); *U* = 627.5, p = 0.17 (active fear behaviour); emergence test, *U* = 607, p = 0. 52 (passive fear behaviour); *U* = 679, p =  0.83(active fear behaviour)). SL chicks tended to perform slightly less comfort behaviours in the open-field test than did SS birds (Mann-Whitney *U*-test, *U* = 545, p = 0.07) (fig. 3). No significant differences in comfort behaviours were observed in the emergence test (Mann-Whitney U-test, U = 688.5, p = 0.92).

Comparisons of numbers of statistically significant differences between chicks' lines for a given adoptive mother's line revealed that control chicks (CL vs CS) differed for 14 parameters, L chicks (LL vs LS) for 7 parameters (and 4 tendencies) and S chicks (SL vs SS) for only 1 parameter (and 1 tendency). So, the effects of adoptive mother's line differed according to chicks' lines (Chi-square test, p<0.005).

## Discussion

We show here, for the first time in bird species, that the young's genetic background modulates its sensitivity to non-genetic maternal influences.

The fearfulness of control-line chicks reared by mothers with a high level of fearfulness was higher than that of chicks reared by females with a low level of fearfulness. C chicks brooded by LTI mothers expressed more fear behaviours when facing a human and in an unfamiliar environment. They also performed a lower level of comfort activities in these situations, these activity levels being inversely correlated to fear level [34]. These data confirm previous results [26] and reveal a strong postnatal influence of mother birds on their chicks' fearfulness, as in mammals [14].

Despite a strong genetic selection, the fearfulness of LTI and STI young was affected by maternal care. LTI chicks brooded by LTI females showed higher fearfulness than did LTI chicks reared by STI mothers: humans and novel environments elicited more fear behaviours. However, adoptive mother's line had no effect on tonic immobility scores and comfort activity levels. SL chicks behaved more fearfully than did SS young only during the hand-on-home-cage-door test, thus revealing only a slight effect of mothering type in the selected STI line. So, the line selected for a low level of fearfulness appeared to be less affected by postnatal maternal influences than the line selected for a high level of emotional reactivity.

Previous reports revealed an impact of genetic factors on rodent mothers' effects. Anisman *et al.* [15] analysed this impact by comparing maternal influence between two strains of mice, one strain showing higher emotional traits, poorer cognitive abilities and less maternal behaviour than the other. Cross-fostering showed that the young of the more emotional strain were strongly influenced by maternal care, presenting especially an increase in their cognitive abilities, whereas the young of the other strain were not influenced. These authors suggested that as the genetic background of the members of the high fearfulness strain could increase their vulnerability to stressor-related disturbances, it would also increase their sensitivity to postnatal maternal influences, and thus enable these organisms to receive the influence of maternal care ‘positively’. Conversely, having a stronger, more stress-resistant genetic background may limit maternal influence and, thereby, may ‘protect’ an organism from possible deficiencies linked to maternal care [15]. These differences of genetic sensitivity to maternal influences in rodents could be linked to inherent differences in the hypothalamic-pituitary-adrenal (HPA) axis activity, but also in the hippocampal synaptic characteristics as the hippocampus plays a major role in the regulation of HPA axis functioning [15]. Liu *et al.* [35] demonstrated that spatial learning by the biological offspring of low LG-ABN (low frequencies of licking/grooming and arched-back nursing) females reared by high LG-ABN mothers were indistinguishable from that by the normal offspring of high LG-ABN mothers. However, the biological offspring of high LG-ABN females reared by low LG-ABN mothers resembled the normal offspring of high LG-ABN mothers. Whereas increased tactile stimulations associated with the presence of a high LG-ABN mother can enhance hippocampal development in low LG-ABN offspring, the higher development at birth of the hippocampus in high LG-ABN offspring reduced their ‘reliance’ on maternal stimulations [35]. Again, the line the least affected by maternal effects is also the line presenting the lowest fearfulness [36].

So, as in rodents, the impact of postnatal maternal influence in quail could be modulated by intrinsic characteristics of the neural system of young, as HPA axis activities in stressful situations differ between LTI and STI birds [37]: LTI chicks' neural system could be very sensitive to maternal tactile stimulations, whereas that of STI young remains highly resistant to this non-genetic maternal influence. However, although maternal tactile stimulations play a fundamental role in the neural and behavioural development of rodent offspring, they cannot be the main source of maternal effects in quail. Indeed, although tactile contacts occur between mother and offspring during warming phases, precocial chicks could also receive many visual and vocal stimulations at an early age. Young birds can learn food preferences [38], maternal behaviour [39] or social behaviour [40] from their mother. So, social learning processes could also be involved in a non-genetic maternal influence mechanism. In our study, for example, chicks may have observed the behaviour of their mothers elicited by humans (experimenter/animal keeper) and so have developed a particular level of fearfulness to humans. As LTI birds express more fear reactions to humans than do STI quail [26], their adopted young, through a learning-by-observation process and also in association with unpleasant physical contacts (scared mothers sometimes tread on chicks), could have develop a higher level of fear of humans than had chicks reared by STI mothers. In this context, differences in sensitivity to maternal influences between the LTI and STI lines could be linked to intrinsic differences in social learning capacities. Few data have explored the cognitive abilities of these two selected lines. Conditioned food aversions were stronger in LTI than in STI quail [41]. However, this difference is modulated by housing and test conditions [41]. In the past two decades, many reports revealed close interactions between emotion and cognition [42], [43]. Although the cognitive abilities of highly emotional animals appeared inferior to those of low emotional animals in various species, recent studies revealed an effect of context and task on the performances evaluated [44]. High stress-reactive mice were better learners than low stress-reactive mice in one task, whereas they underperformed in another task [44]. So, as the cognitive abilities of young could be a potentially strong factor affecting non-genetic maternal influence, especially in precocial species, the cognitive abilities of LTI and STI quail require further investigations, mainly in the context of mothering. Finally, social learning processes imply a motivation and paying attention to conspecifics and their behaviour [45]. Our data showed that the behaviours of STI chick siblings, whatever their mother's line, were less synchronised in the hand-on-home-cage-door test than were LTI chicks. This lower behavioural synchronisation could reflect lower attention of LTI chicks to their cage mates and so lower sensitivity to their environment, thus reducing the effects of maternal care on their development.

In our study, LTI and STI line chicks appeared less sensitive to non-genetic maternal influences than our control line chicks. This result could be an effect of intensive genetic selection. Indeed, a genetic selection over many generations should affect the genetic variability of lines by decreasing heterozigosity [46]. The loss of genetic variability could affect reproductive traits, such as the propensity of individual to mates and the number of offspring in *Drosophila simulans* [47]. Moreover, heterozygous *Drosophila melanogaster* flies appeared more sensitive or more plastic to environmental differences than homozygous flies [48]. So, the genetic selection on tonic immobility duration could have reduced the genetic variability of LTI and STI lines and therefor reduced their sensitivity (or plasticity) to maternal influences compared to control quail.

To conclude, our present data revealed a strong effect of genetic factors on non-genetic maternal influence in birds. The intrinsic behavioural characteristics of a young bird affect strongly its sensitivity to environmental influences and so play a fundamental role in behavioural development processes. Our study also revealed strong similarities between birds and mammals (especially rodents) although the development of young, maternal care and relationship with mother are very different. Although studies on mammals have revealed some neurobiological mechanisms explaining genetic effects, birds, and especially quail (precocial species), are also good models to analyse behavioural mechanisms, for instance, attentional processes or cognitive abilities, during mothering phase.
